# Contrasting Effects of an Atherogenic Diet and High-Protein/Unsaturated Fatty Acids Diet on the Accelerated Aging Mouse Model SAMP8 Phenotype

**DOI:** 10.3390/neurolint16050080

**Published:** 2024-09-23

**Authors:** Jesús Llanquinao, Claudia Jara, Daniela Cortés-Díaz, Bredford Kerr, Cheril Tapia-Rojas

**Affiliations:** 1 Laboratory of Neurobiology of Aging, Centro de Biología Celular y Biomedicina (CEBICEM), Facultad de Medicina y Ciencia, Universidad San Sebastián, Providencia-Santiago 7510157, Chile; jllanquinaos@correo.uss.cl (J.L.); cjara@cienciavida.org (C.J.); dcortesd1@correo.uss.cl (D.C.-D.); 2 Laboratory of Neuroendocrinology and Metabolism, Centro de Biología Celular y Biomedicina (CEBICEM), Facultad de Medicina y Ciencia, Universidad San Sebastián, Providencia-Santiago 7510157, Chile; 3 Centro Científico y Tecnológico de Excelencia Ciencia & Vida, Fundación Ciencia & Vida (FCV), Avenida Del Valle Norte #725, Huechuraba, Santiago 8580702, Chile

**Keywords:** mitochondrial function, diets, SAMP8 mice, cognitive function, hippocampus

## Abstract

**Background/Objectives:** Aging has been extensively studied, with a growing interest in memory impairment by a neurobiological approach. Mitochondrial dysfunction is a hallmark of aging, contributing to the aging phenotype; therefore, mitochondrial interventions seem fundamental. The diet is a physiological approximation for modifying mitochondria, which could impact the age-related phenotype. **Methods:** We studied two diets with low-carbohydrate and high-fat compositions, differing in the amount of protein and the fat type disposable—the atherogenic diet Cocoa (high protein/high saturated fat/high cholesterol) and the South Beach diet (very high-protein/high-unsaturated fat)—on oxidative stress, mitochondrial state, and hippocampus-dependent memory in 3-month-old Senescence-Accelerated Mouse Model (SAMP8) seed over 3 months to determine their pro- or anti-aging effects. **Results:** Despite its bad reputation, the Cocoa diet reduces the reactive oxygen species (ROS) content without impacting the energy state and hippocampus-dependent spatial acuity. In contrast to the beneficial impact proposed for the South Beach diet, it induced a pro-aging phenotype, increasing oxidative damage and the levels of NR2B subunit of the NMDA, impairing energy and spatial acuity. Surprisingly, despite the negative changes observed with both diets, this led to subtle memory impairment, suggesting the activation of compensatory mechanisms preventing more severe cognitive decline. **Conclusions:** Our results demonstrated that diets usually considered good could be detrimental to the onset of aging. Also, probably due to the brain plasticity of non-aged animals, they compensate for the damage, preventing a more aggravated phenotype. Nevertheless, these silent changes could predispose or increase the risk of suffering pathologies at advanced age.

## 1. Introduction

Aging is often accompanied by changes in lipid metabolism and an increased risk of cardiovascular disease [1]. The consumption of diets high in saturated fats and cholesterol, commonly called atherogenic diets, is implicated in the development of cardiovascular diseases. The consumption of an atherogenic diet exacerbates these age-related changes, leading to the accelerated progression of atherosclerosis and increased risk of cardiovascular events [2]; however, the link to accelerating brain aging in the absence of pathology is unclear. An atherogenic diet exacerbates oxidative stress by increasing the production of ROS and impairing antioxidant defenses in peripherical tissues. Rodents fed an atherogenic diet show increased oxidative stress in the vasculature and other tissues, contributing to endothelial dysfunction and accelerated aging [3,4]. However, studies suggest that these diets may also affect the brain, particularly memory formation-related processes and mitochondrial function. Middle-aged rats (16 months old (mo)) fed a high-fat/high-cholesterol diet for 8 weeks exhibited impairment of spatial memory, accompanied by a loss of dendritic integrity [5]. Similarly, 5 mo rats fed saturated fats for 8 weeks showed an impairment of the hippocampus without affecting other brain regions that are important for learning [6]. Also, evidence suggests that an atherogenic diet may damage mitochondria in the brain, potentially contributing to cognitive decline. Mice fed a high-fat diet showed increased oxidative stress and neuroinflammation in the hippocampus, accompanied by mitochondrial dysfunction [7,8]. These alterations were associated with deficits in spatial memory and synaptic plasticity [9]. Thus, the consumption of an atherogenic diet may have detrimental effects on the brain, which has been linked to an increased risk of neurodegenerative diseases, possibly accelerating the aging phenotype; however, the latter has not been tested.

In contrast, dietary patterns rich in protein and unsaturated fats have garnered attention for potential health benefits, including brain function. Animal studies also have investigated the cognitive effects of this diet on memory. Rats fed a diet enriched with omega-3 fatty acids, a type of unsaturated fat, showed improved spatial learning and memory [10]; similarly, 19 mo C57Bl/6J male mice fed for 8 weeks with a polyunsaturated fatty acid showed improved hippocampus-dependent recognition and spatial and localization memories [11]. Human studies have also provided insights into the cognitive effects of diets rich in unsaturated fats and protein. A randomized controlled trial found that older adults consuming a Mediterranean-style diet supplemented with olive oil, a source of unsaturated fats, showed improved cognitive function, including memory [12]. Moreover, a cross-sectional study observed a positive association between a higher intake of unsaturated fats and better memory performance in older adults, slower rates of episodic memory decline, and an early risk factor for AD [13].

Also, evidence suggests that diets rich in unsaturated fats and protein may influence mitochondrial function in the brain. Observational studies in humans have suggested a link between adherence to a Mediterranean-style diet, high in unsaturated fats and protein sources such as fish and nuts, and the preservation of mitochondrial function with aging [14,15]. Animal studies have shown that supplementation with omega-3 fatty acids enhances mitochondrial biogenesis and function in the hippocampus, a brain region critical for memory [16]. Moreover, a high-protein diet increased brain mitochondrial respiration and ATP production in models of neurodegeneration and Parkinson’s disease [17,18]. Thus, considering these antecedents, a diet rich in protein and unsaturated fats will improve the health state of adult individuals and reduce cognitive and mitochondrial failure in neurodegenerative diseases; however, if this diet reduces the appearance of the aging phenotype has yet to be explored.

Here, we fed 3 mo Senescence-Accelerated Mouse Model (SAMP8) mice with two distinct diets with low-carbohydrate and high-fat compositions, differing in the fat type disposable—(1) the atherogenic diet Cocoa, which is high protein, high saturated fat, and high cholesterol and (2) the South Beach diet, which is composed of very high levels of protein and high unsaturated fat—to probe their impacts on the onset of brain aging, particularly on the redox and bioenergetic states of the hippocampal brain and the spatial memory. Surprisingly, our results reveal that the South Beach diet increases oxidative damage and the protein levels of the excitotoxicity-related NR2B subunit of NMDA and decreases the ATP content, finally reducing the spatial acuity to learn and memorize a spatial task. However, this is accompanied by increased levels of fission and fusion mitochondrial proteins and antioxidant enzymes and an increment in pre- and post-synaptic proteins, suggesting the participation of compensatory mechanisms that could counteract the damage, including mitochondrial fission and fusion, antioxidant response, and synaptic plasticity. In contrast, the Cocoa diet reduces the ROS content, suggesting a positive effect without deleterious effects on oxidative, mitochondrial, energy, and synaptic protein states and no significant impact on hippocampal function measuring the spatial acuity. Thus, our results suggest that a potentially beneficial diet will be detrimental to the onset of aging. Nevertheless, this damage is not more severe, possibly due to the activation of compensatory mechanisms. Finally, these minor changes following the diet will be considered predisposing or risk factors for developing age-related pathologies. More studies are necessary to validate this proposal.

## 2. Materials and Methods

### 2.1. Animals

SAMP8 mice (Senescence-Accelerated Mouse Prone 8) that were 3 months old were handled according to National Institutes of Health (NIH, Baltimore, MD) guidelines. The animals were housed in a temperature-controlled room (24 °C) on a 12 h light/dark cycle, with food and water ad libitum. The Bioethics and Biosafety Committee of the Universidad San Sebastián, Chile, approved all experimental procedures described here. After the behavior test, the animals were euthanized. Then, the hippocampus was removed for biochemical analysis, with n = 3 animals per group. To determine the cognitive differences and mitochondrial bioenergetic function after diet treatments, each group was formed by n = 6 animals.

### 2.2. Mice Treatment

Three-month-old SAMP8 mice were divided into three groups: Control diet (14% fat, 25.9% protein, and 60% carbohydrate; Prolab PMH3000, code 5P00), Cocoa diet (56.5% fat, 33.6% protein, and 9.9% carbohydrate; test diet, code 57BB), and South Beach diet (38.8% fat, 47.5% protein, and 13.6% carbohydrate; test diet, code 5TVW). The diet treatment began when the animals were 3 months old. All groups finished treatment at 6 months old. All animals were fed the Control diet from weaning until 3 months. Their body weights and daily diet consumption were recorded for 3 months.

The Control mouse diet generally contains a balanced mix of fatty acids, including saturated fats, monounsaturated fats (MUFAs), polyunsaturated fats (PUFAs), and cholesterol. The approximate values are as follows: saturated fats: about 1–2% of the total diet; MUFAs: about 1–2% of the total diet; PUFAs: about 5–6% of the total diet; cholesterol: typically very low, often less than 0.1% of the total diet [19,20,21]. The Cocoa diet is characterized by its high fatty acid content from cocoa butter. Also, it is rich in saturated fatty acids, such as palmitic acid, MUFAs, and glucose [22]. The South Beach diet is hyperproteic and is mainly composed of casein, glutamic acid, and leucine, and it has a high level of PUFAs [23].

### 2.3. Behavioral Tests

The Morris Water Maze (MWM) behavioral test was monitored using Any-MAZE Behavioral software, 7.45 version (Stoelting Co., Wood Dale, IL, USA), using cameras and instruments manufactured or recommended by the manufacturer. The MWM behavioral test was conducted during the 12 h light phase of the animals’ light/dark cycle, as previously described [24]. The mice were trained in a 1.2 m diameter circular pool (19–24 °C, water). Each animal was trained on the location of the platform. The test was performed for ten consecutive days, with three daily trials, except days 6 and 7 (training off). A 9 cm submerged platform was used, with a maximum trial duration of 60 s, where each mouse was introduced into the pool from the opposite quadrant of the platform. The test was performed thrice daily, and the escape latency time required to reach the platform as well as the time spent in each quadrant were measured. The platform was removed 24 h after the last training, and we evaluated the time each animal remained in the platform area for 1 min. For the spatial acuity parameter, the pool was subdivided by imaginary lines into four equal quadrants. These lines, in turn, intersected the edge of the pool at the arbitrary cardinal start locations named north, south, east, and west. Furthermore, the pool was divided into three equidistant concentric annuli or zones. The software obtained the percentage of permanence in specific regions of the pool. Then, the product of the percentage of permanence in the platform quadrant by the percentage of permanence in the platform zone was calculated.

### 2.4. Reagents and Antibodies

Lysis buffer was prepared (HEPES buffer pH 7.4, NaCl 0.5 M, EDTA 1 mM, EGTA 1 mM, NP-40 1%, and NaF 25 mM, supplemented with protease and phosphatase inhibitors). A BCA Protein Assay Kit (23227, Thermo Fisher Scientific, Waltham, MA, USA), and an ATP determination kit (A22066, Invitrogen, Waltham, MA, USA) were acquired. The primary antibodies used were as follows: mouse anti-actin (1:1000, sc-1616, Santa Cruz Biotechnology, Inc., Dallas, TX, USA), rabbit anti-Arc (1:1000, cat N°156003, Synaptic System, Göttingen, Germany), mouse anti-ATP5A (1:1000, sc-136178, Santa Cruz Biotechnology, Inc., Dallas, TX, USA), mouse anti-catalase (1:1000, sc-271803, Santa Cruz Biotechnology, Inc., Dallas, TX, USA), mouse anti-DRP1 (1:1000, sc-271583, Santa Cruz Biotechnology, Inc., Dallas, TX, USA), rabbit anti-p616DRP1 (1:1000, mAb 6319; mb 4494, Cell Signaling Technologies, Freeport, TX, USA), mouse anti-GluR1 (1:1000, sc-13152, Santa Cruz Biotechnology, Inc., Dallas, TX, USA), mouse anti-glutathione reductase (1:1000, sc-133245, Santa Cruz Biotechnology, Inc., Dallas, TX, USA), mouse anti-HOMER (1:1000, sc-17842, Santa Cruz Biotechnology, Inc., Dallas, TX, USA), mouse anti-4-hydroxy-2-nonenal (4-HNE) (1:1000, 298112, US Biological, Salem, MA, USA), rabbit anti-MFN2 (1:1000, mAb 11925, Cell Signaling Technologies, Beverly, MA, USA), rabbit anti-MFF (1:1000, mAb 84580, Cell Signaling Technologies, Beverly, MA, USA), mouse anti-Nr2b (1:1000, sc-365597, Santa Cruz Biotechnology, Inc., Dallas, TX, USA), mouse anti-Nrf2 (1:1000, sc-365949, Santa Cruz Biotechnology, Inc., Dallas, TX, USA), mouse anti-OSCP (1:1000, sc-365162, Santa Cruz Biotechnology, Inc., Dallas, TX, USA), rabbit anti-OPA1 (1:1000, mAb 80471, Cell Signaling Technologies, Beverly, MA, USA), mouse anti-Total OXPHOS Human WB antibody cocktail (1:1000, ab110411, Abcam, Inc., Cambridge, UK), mouse anti-PGC-1α (1:1000, sc-517380, Santa Cruz Biotechnology, Inc., Dallas, TX, USA), mouse anti-PSD95 (1:500, sc-32290, Santa Cruz Biotechnology, Inc., Dallas, TX, USA), mouse anti-synapsin (1:1000, sc-376623, Santa Cruz Biotechnology, Inc., Dallas, TX, USA), mouse anti-synaptophysin (1:1000, sc-17750, Santa Cruz Biotechnology, Inc., Dallas, TX, USA), mouse anti-TOM40 (1:1000, sc-365467, Santa Cruz Biotechnology, Inc., Dallas, TX, USA), mouse anti-⍺-Tubulin (1:1000, Cat 32–2500, Invitrogen, Waltham, MA, USA), and mouse anti-VDAC (1:1000, sc-390996, Santa Cruz Biotechnology, Inc., Dallas, TX, USA). The fluorescent dye used was CM-H2DCFDA (catalog number C6827, Thermo Fisher Scientific, Waltham, MA, USA).

### 2.5. Measurement of ROS Content

The ROS content was measured using the fluorescent dye CM-H2DCFDA (catalog number C6827, Thermo Fisher Scientific, Waltham, MA, USA), as previously described [24]. Briefly, hippocampal lysates were diluted in respiration buffer and added to a black 96-well plate followed by 25 μM DCF. The plate was then incubated for 5 min at room temperature. After this time, the ROS content was measured on the BioTek Synergy HT (485 nm; 530 nm).

### 2.6. Measurement of ATP Content

The ATP concentration was measured in the obtained hippocampal tissue lysate using a luciferin/luciferase bioluminescence assay kit (ATP determination kit N° A22066, Molecular Probes, Invitrogen), as previously described [25]. The amount of ATP in each diet treatment was calculated from standard curves and normalized to the total protein concentration.

### 2.7. Immunoblotting

Hippocampus from 6 mo mice were dissected on ice and immediately processed, as previously described (n = 3) [25]. Briefly, hippocampal tissue was homogenized in HEPES buffer (25 mM HEPES, 125 mM NaCl, 25 mM NaF, 1 mM EDTA, 1 mM EGTA, and 1% NP-40, pH = 7.4) supplemented with a mixture of protease inhibitors (catalog number 78429, Thermo Fisher Scientific) and phosphatase inhibitors (NaF 50 mM, Na_2_P_2_O7 334 µM, and Na_3_VO_4_ 1 mM) using a homogenizer and then passed sequentially through syringes of different gauges. The protein samples were centrifuged at 14,000 rpm for 20 min at 4 °C. The protein concentrations were determined using the BCA protein assay kit (catalog number 23225, Pierce, Rockford, IL, USA). Samples were resolved using SDS-PAGE, followed by immunoblotting on PVDF or nitrocellulose membranes. The membranes were incubated with the primary antibody and peroxidase-conjugated mouse or rabbit IgG antibodies (Pierce) and visualized with an ECL kit (Luminata Forte Western HRP substrate, Millipore).

### 2.8. Statistical Analysis

The results are presented as bar graphs indicating the mean ± SEM. Statistical significance was determined using one-way ANOVA with Bonferroni’s post-test. *p*-values > 0.05 and ≤0.05 were regarded, respectively, as not statistically significant and as statistically significant. In the figures, *p*-values between 0.01 and 0.05 are marked with one asterisk, *p*-values between 0.001 and 0.01 are with two asterisks, *p*-values less than 0.001 are shown with three asterisks, and *p*-values less than 0.0001 are shown with four asterisks. Minor *p*-values of 0.1 (*t*-test with 90% confidence interval) are marked with one hash mark (#). All statistical analyses were performed using Prism software 8 (GraphPad Software, Inc., Boston, MA, USA).

## 3. Results

### 3.1. The Cocoa Diet Increases the Body Weights of SAMP8 Mice

The 3 mo SAMP8 mice were divided into three groups and fed (i) a Control diet, (ii) the Cocoa diet, and (iii) the South Beach diet for three months to assess whether the diets promoted a pro- or anti-aging effect. We define the Cocoa diet as atherogenic because its main ingredients are 7.5% cocoa butter, 7.5% casein, and 1.25% cholesterol. In addition, its central energy intake comes from fatty acids (56.5%), its nutritional profile is 15.5%, and it is high in casein and cholesterol [26]. In contrast, the South Beach diet is a very high-protein and high-unsaturated fat diet, with a nutritional composition of 52% protein, 18.9% fatty acids, of which 9% are PUFAs, and 14.9% carbohydrates. The ingredients with the highest contents are casein vitamin (58.01%), corn starch (10.72%), and soybean and corn oils (13.98%). In addition, its central energy intake is obtained from protein (47.5%). This comparison was performed because different effects can be observed when comparing two diets with similar proportions of carbohydrates and fat but with varying amounts of proteins and other types of fat. Not all fats have the same effect on health. For example, unsaturated fats can help decrease inflammation that is linked to several chronic diseases [27]. In contrast, saturated fatty acids induce inflammation in obesity and other pathologies [28]; however, their effects on age-related phenotypes are unclear.

**Figure 1:**
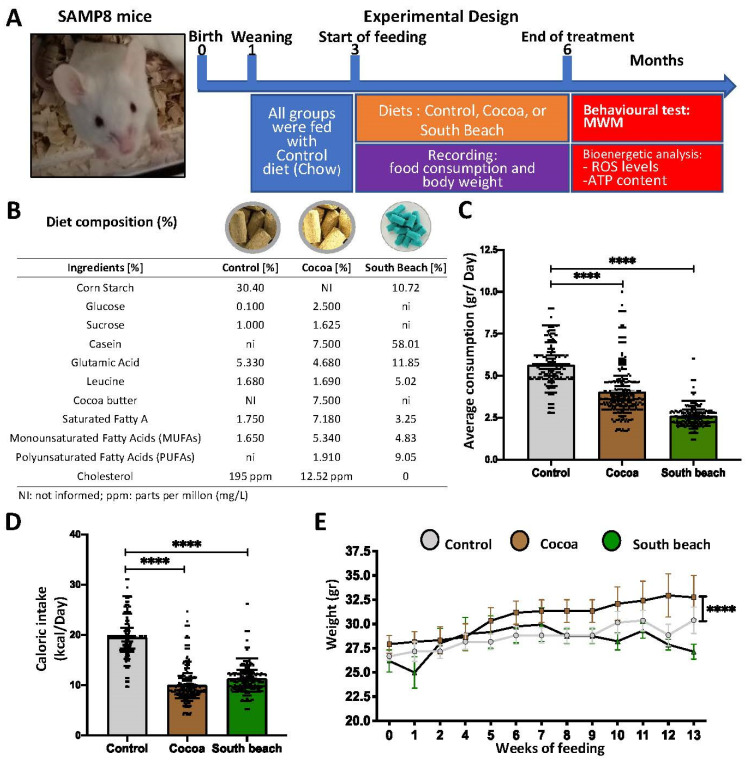
The Cocoa and South Beach diets reduce food and calorie intakes, and the Cocoa diet induces weight gain in SAMP8 mice. (**A**) Schematic representation of the experimental procedure for SAMP8 mice divided into three groups—1: Control diet; 2: Cocoa diet; and 3: South Beach diet—for three months of the dietary pattern. Behavioral tests and biochemical assays were performed at 6 mo. (**B**) The composition of the experimental diets. (**C**) Daily consumption of the experimental diets. (**D**) Caloric intake is calculated by the formulation of each of the diets. (**E**) Body weight records for 13 weeks for each experimental group. Graph bars represent means ± SEM. N = 6 animals per treatment group with the experimental diets. One-way ANOVA and repeated measures ANOVA with Bonferroni’s post-test. **** *p* < 0.0001.

Following the diet protocol at 6 mo, before developing a brain-aging phenotype characterized by oxidative damage, mitochondrial and synaptic failures, and memory loss (commonly observed from 7 mo), we evaluated the spatial memory in the Morris Water Maze (MWM) test. Then, the redox, mitochondrial, and synaptic states were evaluated using biochemical assays, as described in Figure 1A. Each diet was administered ad libitum, and the compositions of the experimental diets are indicated in Figure 1B. In our study, we observed a reduction in the average daily intake of mice fed the Cocoa and South Beach diets (Figure 1C) and caloric intake (Figure 1D). SAMP8 mice fed the Cocoa diet, but not the South Beach diet, increased their body weight compared to SAMP8 mice fed the Control diet (Figure 1E). Thus, our results indicate that SAMP8 mice fed the Cocoa and South Beach diets reduced their food and caloric intakes. Still, despite this, the Cocoa diet increases body weight, suggesting an obesogenic effect.

### 3.2. The South Beach Diet Has an Enhanced Level of Oxidative Damage

**Figure 2:**
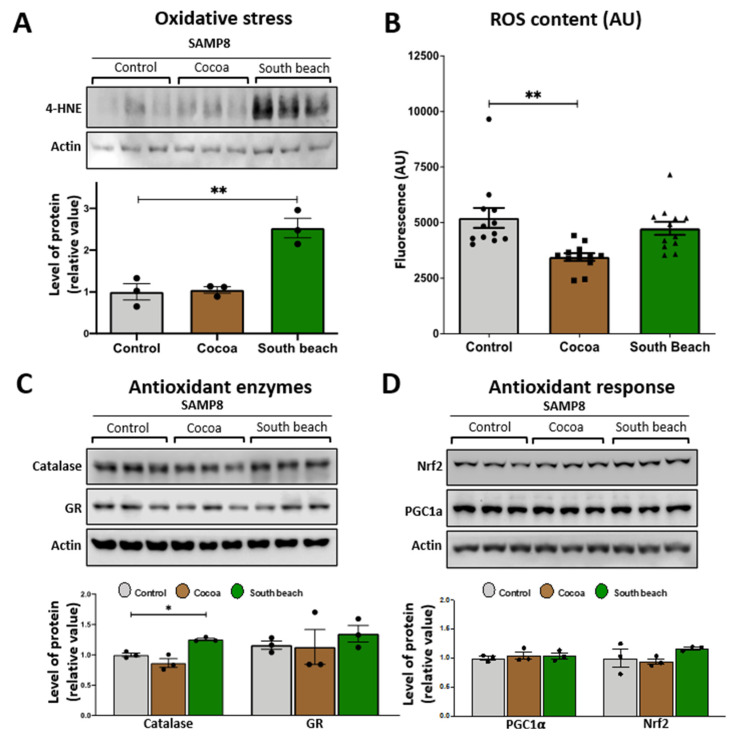
Effect of 12 weeks of the Cocoa and South Beach diets on the level of oxidative damage in the hippocampus of SAMP8 mice. (**A**) Western blot and densitometric analysis of hippocampal lysates for oxidative damage with antibodies against 4-hydroxynonenal (4-HNE). (**B**) ROS content in the whole hippocampal lysate using the fluorescent dye CM-H2DCFDA. Western blot and densitometric analysis for (**C**) antioxidant enzymes and (**D**) antioxidant response mediators in the hippocampus of SAMP8 mice exposed to Control, Cocoa, or South Beach diets. Graph bars represent means ± SEM. N = 3 animals per group; N = 6 animals per group for ROS content. One-way ANOVA with Bonferroni’s post-test. * *p* < 0.05; ** *p* < 0.01.

Mitochondria are organelles that require the maintenance of redox balance. The damage to mitochondrial function increases reactive oxygen species (ROS) production [29]. We evaluated the levels of oxidized proteins using an anti-4-HNE antibody that detects the stably formed HNE–protein adducts (products of lipid peroxidation) [30]. Our results showed that the levels of oxidized proteins were significantly higher in the hippocampus of the mice fed with the South Beach diet for three months than those fed with the Control diet. In contrast, the Cocoa diet did not affect the levels of oxidized proteins (Figure 2A). Then, we evaluated the levels of ROS content in the hippocampal lysate, and we observed that the ROS content showed significant differences between the Control and Cocoa diet-fed mice. Specifically, the levels of ROS were significantly reduced in the hippocampus of the Cocoa diet-fed mice, suggesting a healthier ROS state after this dietary pattern, without showing changes in the ROS content in the South Beach diet group (Figure 2B).

In addition, and considering that oxidative damage occurs due to an imbalance between ROS production and oxidative defenses [31], we measured the levels of the antioxidant enzymes catalase and glutathione reductase (GR). Our results show that only South Beach diet-fed mice showed a significantly increased level of the antioxidant enzyme catalase compared with the Control diet-fed mice. In contrast, the GR levels were unchanged after both diet-feeding conditions (Figure 2C). It is known that increased oxidative damage induces the activation of signaling pathways to activate an antioxidant [32]. For this, we evaluated the protein levels of two antioxidant signaling mediators, Nrf2 and PGC-1α (Figure 2D). We observed that the PGC-1α and Nrf2 levels were similar between all groups. In summary, our results show that the high-protein/high-saturated and polysaturated-fat/high-cholesterol Cocoa diet reduced the ROS content and did not evoke any change in protein oxidative damage. In contrast, the high-protein, high-unsaturated fat South Beach diet induces oxidative damage even though antioxidant defenses seem to be active, increasing the levels of the antioxidant enzyme catalase in the hippocampus of SAMP8 mice. This last finding could be a compensatory mechanism against the increment of oxidized proteins to reduce the oxidative stress induced by the South Beach diet.

### 3.3. The South Beach Diet Increases the Levels of Proteins Involved in Mitochondrial Fusion and Fission, Suggesting Increased Mitochondrial Dynamic Processes in the Hippocampus of SAMP8 Mice

**Figure 3:**
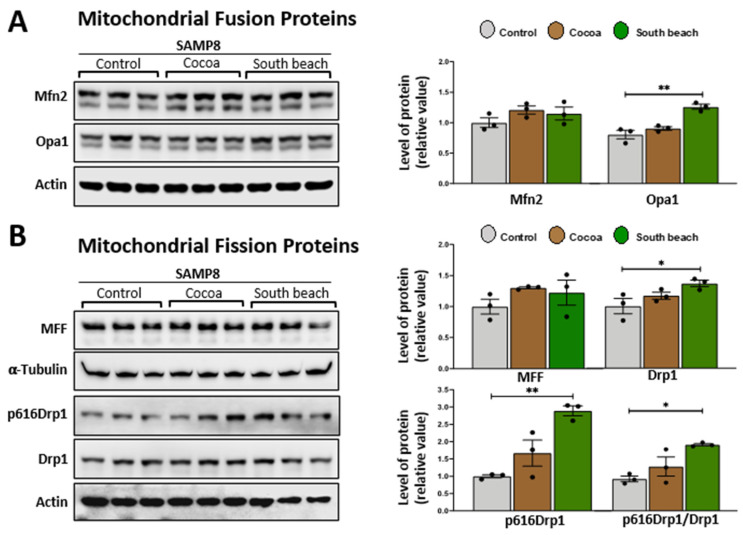
The South Beach diet increases proteins involved in mitochondrial fusion and fission processes in the hippocampus of SAMP8 mice. Western blot and densitometry analysis of (**A**) the fusion proteins Mfn2 and Opa1 and (**B**) the mitochondrial fission proteins MFF, Drp1, and phosphorylated Ser616 Drp1 in the hippocampal lysates of SAMP8 mice fed Control, Cocoa, and South Beach diets. Graph bars represent means ± SEM. N = 3 animals per group. One-way ANOVA with Bonferroni’s post-test. * *p* < 0.05; ** *p* < 0.01.

Mitochondrial fusion and fission processes are crucial in maintaining mitochondrial integrity and function, and critical proteins mediate them [33]. Dynamin-related GTPase mitofusins (Mfn1 and Mfn2) and optic atrophy 1 (Opa1) are responsible for the fusion of the outer and inner mitochondrial membranes. In contrast, dynamin-related protein 1 (Drp1) is recruited to the outer mitochondrial membrane to constrict mitochondria, inducing its division, and its phosphorylation at Ser616 stimulates this process [34], with MFF (mitochondrial fission factor) as an essential factor for mitochondrial recruitment of Drp1 [35]. Previously, it was reported that 3 mo rats fed diets rich in saturated fatty acids had increased mitochondrial fission processes, whereas the polyunsaturated fatty acid diet increased mitochondrial fission processes in hepatocytes [36]. However, the effects of the Cocoa and South Beach diets on mitochondrial fusion/fission proteins in the hippocampus have not been described. We observed that mice fed the South Beach diet, but not the Cocoa diet, had significantly increased protein levels of Opa1 (Figure 3A), Drp1, and its phosphorylation in Ser616 (Figure 3B). These results suggest that the high-protein/unsaturated fat South Beach diet stimulates the fission and fusion processes in the hippocampus of SAMP8 mice, possibly to reduce the oxidative effect of this diet, which is rich in protein and unsaturated fatty acids, or other mitochondrial alterations.

### 3.4. The Cocoa and South Beach Diets Reduce the Levels of an Essential Protein Implicated in Energy Production, but Only the South Beach Diet Reduced the Energy State in the Hippocampus of SAMP8 Mice

**Figure 4:**
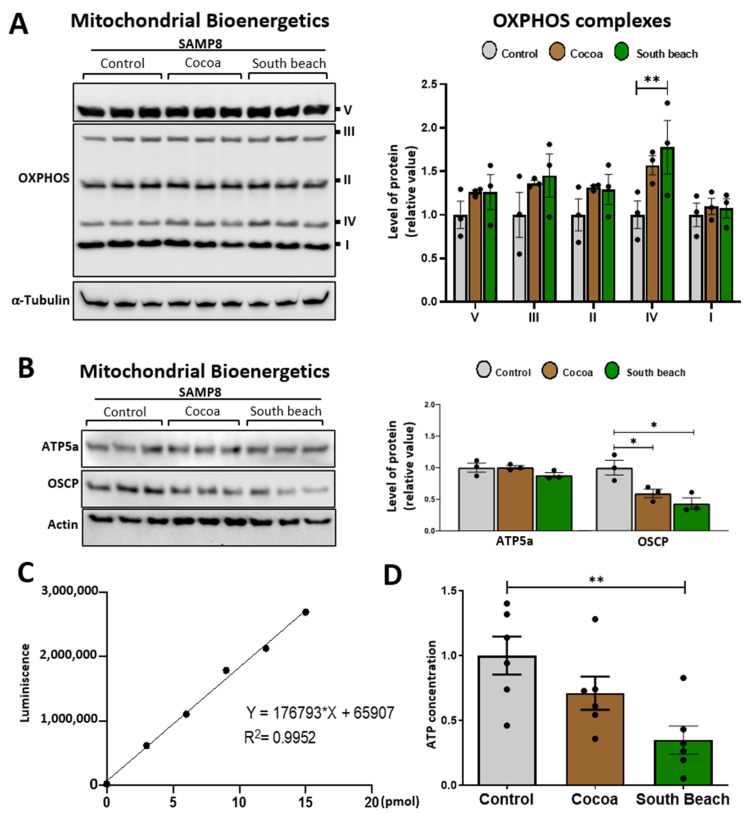
The South Beach diet reduced the energy state in the hippocampus. (**A**) Western blot and densitometric analysis for mitochondrial oxidative phosphorylation (OXPHOS) complexes I–V in whole hippocampal extracts. (**B**) Western blot and densitometric analysis of mitochondrial ATP synthase subunits ATP5A and OSCP. (**C**,**D**) ATP content in the hippocampus of mice. Graph bars represent means ± SEM. N = 3 animals per group for Western blot. N = 6 animals per group for ATP content. One-way ANOVA with Bonferroni’s post-test. * *p* < 0.05; ** *p* < 0.01.

Mitochondria are the principal producers of ATP in neurons, and during the aging process, the energy states are specifically susceptible to damage [30]. Thus, we evaluated the impact of diet on the mitochondrial bioenergetics of the hippocampus of SAMP8 mice. We assessed the protein levels of the mitochondrial respiratory complexes involved in oxidative phosphorylation (OXPHOS) using the antibody cocktail OXPHOS, which contains a mix of antibodies specific to the five mitochondrial complexes [30,37]. We observed that mice from all feeding groups had similar levels of all OXPHOS complexes (Figure 4A). Next, we evaluated whether feeding with these diets affects the levels of the proteins necessary for mitochondrial bioenergetics production by the ATP synthase. We evaluated ATP5A, the catalytic subunit of the mitochondrial ATP synthase complex responsible for the synthesis/hydrolysis of ATP [38], and the synthase oligomycin sensitivity-conferring protein (OSCP) subunit, which ensures efficient energy interconversion to form ATP [39,40]. We observed similar ATP5A levels in the hippocampus of mice fed the different diets and significantly decreased levels of OSCP in the hippocampus of mice fed the Cocoa and South Beach diets compared to the Control diet (Figure 4B). The OSCP decrease is part of mitochondrial dysfunction and associates cognitive aging with dementia and neurodegenerative diseases [40]. Interestingly, when we evaluated ATP production, we observed that the hippocampus of mice fed the Control diet had significantly higher ATP levels than those fed the South Beach diet (Figure 4C,D). Therefore, our results indicate that feeding with the South Beach diet, and possibly also with the Cocoa diet, reduces the energy state in the hippocampus of SAMP8 mice and promotes the aging phenotype.

### 3.5. The South Beach Diet Increases the Levels of Synaptic Proteins, Suggesting Excitotoxicity in the Hippocampus of SAMP8 Mice

**Figure 5:**
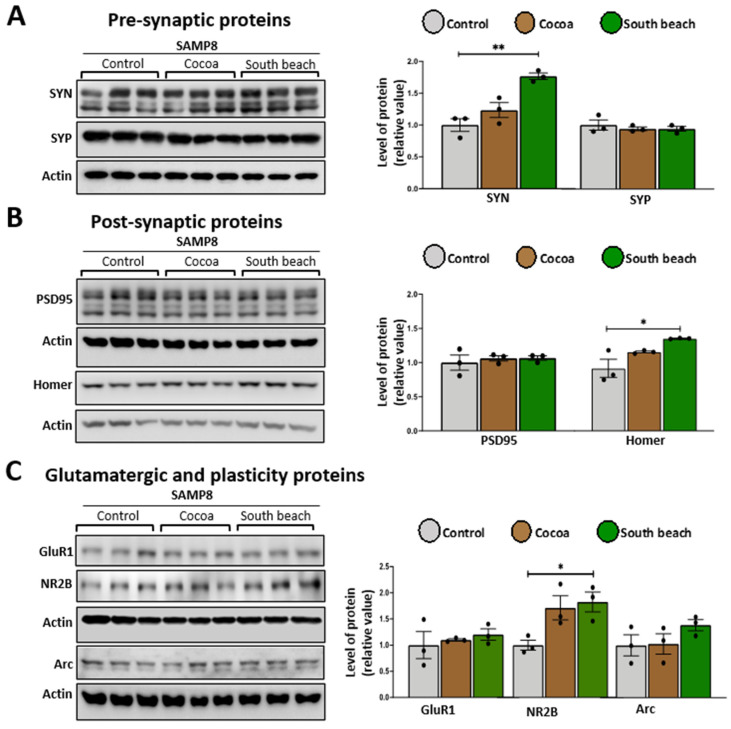
The South Beach diet up-regulated pre-synaptic, post-synaptic, and glutamatergic receptor subunits in SAMP8 mice. Western blot of hippocampal lysates measured the levels of (**A**) pre-synaptic proteins, (**B**) post-synaptic proteins, and (**C**) glutamatergic ionotropic receptor subunits and their respective densitometric analysis. Graph bars represent means ± SEM. N = 3 animals per group. One-way ANOVA with Bonferroni’s post-test. * *p* < 0.05; ** *p* < 0.01.

Mitochondrial function is crucial for maintaining synaptic function due to ROS signaling, Ca^2+^ buffering, and the high energy demand of neuronal cells, including synthesizing synaptic proteins and the assembly of scaffolding proteins and receptors [41]. During aging, synaptic proteins are dysregulated [42], and different diets can modulate them [43], so we evaluated structural pre-synaptic and post-synaptic proteins and glutamatergic receptor subunits using a Western blot in the protein extracted from the hippocampus of mice fed the different diets. As pre-synaptic proteins, we measured synapsin (SYN), which modulates neurotransmitter release by reversibly tethering synaptic vesicles to the actin cytoskeleton [44], and synaptophysin (SYP), which is necessary for synaptic vesicle recycling [45]. To evaluate the post-synaptic structure, we determined the levels of the scaffold proteins PSD95 and Homer, the principal regulators of excitatory synapses, by interacting with AMPA and NMDA receptors [46], and the protein binding to metabotropic glutamate, inositol trisphosphate and ryanodine receptors, and Shank family proteins, respectively. Finally, to assess excitatory synapsis, we measured the levels of subunits ionotropic receptor AMPA and NMDA GluR1 and NR2B, respectively. We observed that in the hippocampus of the Cocoa diet-fed mice, there was no change in the levels of any measured proteins (Figure 5). In contrast, in the hippocampus of the South Beach diet-fed mice, pre-synaptic protein SYN (Figure 5B), scaffold protein Homer (Figure 5B), and the receptor subunit NR2B (Figure 5C) are up-regulated. Thus, our results suggest that the South Beach diet promotes increased synaptic activity; however, considering that the NR2B subunit is involved in NMDA-mediated excitotoxicity [47], this could be related to a negative effect on the synaptic function and could affect cognitive processes as observed in aging and age-related diseases.

### 3.6. Cocoa and South Beach Diets Show Reduced Hippocampus-Dependent Spatial Acuity

**Figure 6:**
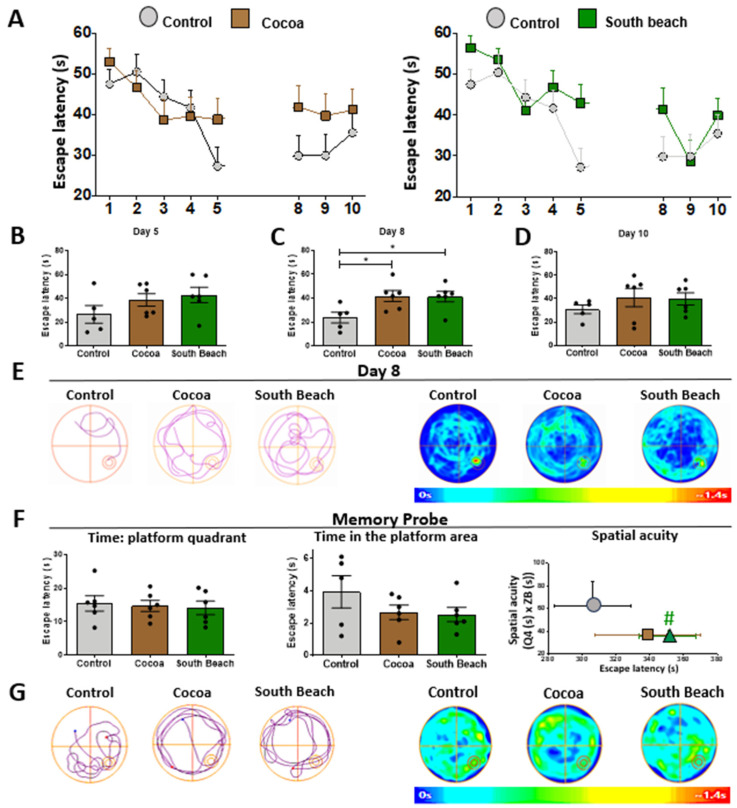
Hippocampal learning and memory impairment are observed in animals fed the Cocoa and South Beach diets. (**A**) Escape latency during the Morris Water Maze test. Statistical analysis of training days: (**B**) day 5, (**C**) day 8, and (**D**) day 10. (**E**) Representative track plot and heat maps of one animal per group on day 8. (**F**) Time each group spent in the platform area, the number of entries to the platform area during the probe test, and the spatial acuity. (**G**) Tracks of the plot and heat maps of each group in the probe test. Graph bars represent means ± SEM. N = 5 for the Control diet and 6 animals each for the Cocoa and South Beach diets. One-way ANOVA with Bonferroni’s post-test. * *p* < 0.05; # *p* < 0.1 *t*-test, 90% confidence interval.

Because different diets can modulate brain function [43], the Cocoa and South Beach diet feedings could affect the hippocampus of SAMP8 mice, such as by causing memory impairment. Therefore, we evaluated hippocampus-dependent spatial learning and memory capacity through the most commonly used test to assess this type of memory, the Morris Water Maze (MWM) behavioral test [48]. In this test, each mouse was placed thrice daily in a pool to find the hidden escape platform, being guided by spatial cues for ten days. We observed that both diet-feeding groups took longer to find the hidden platform during training days (Figure 6A,B). This phenomenon is significantly different on the first day of memory consolidation (day 8), where the Cocoa and South Beach diet-fed mice had difficulty learning the location of the hidden platform, in contrast to the Control diet-fed mice (Figure 6C–E). However, on the test day, when the platform was out, their spatial memory was assessed, and the South Beach diet-fed mice tended to spend less time in the platform area than the Control mice (Figure 6F,G). Finally, we measured spatial acuity, a more sensitive parameter to evaluate spatial learning, by representing the probability of finding the mice in a specific region around the hidden platform. As observed in the graph (Figure 6C, right), the mice fed the Cocoa or South Beach diets are localized in a region of the graph with high escape latency values and low spatial acuity scores, reflecting the impaired spatial memory of these animals. These changes are significant in the South Beach diet group. Thus, these results suggest that a high-fat (Cocoa) and high-protein (South Beach) diet impairs hippocampal learning and memory ability in SAMP8 mice and that the South Beach diet also reduces spatial acuity, promoting an aging phenotype and predisposing them to develop future age-related pathologies.

## 4. Discussion

SAMP8 mice are an animal model for studying aging and early-onset hippocampus-dependent cognitive impairment, and this aging phenotype typically manifests in our and other colonies after 7 mo [49,50]. In this study, we investigated the effects of two types of diets: Cocoa (56.5% fat; 33.6% protein; 9.9% carbohydrate) and South Beach (38.8% fat, 47.5% protein, and 13.6% carbohydrate) compared with the Control diet (14% fat; 25.9% protein; 60% carbohydrate) on oxidative stress, mitochondrial and energetic states, synaptic proteins, and cognitive performance to elucidate their pro- or anti-aging effects. Our results reveal that feeding with the atherogenic diet Cocoa, which is high protein, high saturated and polyunsaturated fats, and high cholesterol, has minimal effects, with unchanged oxidative damage and mitochondrial proteins, cell energy state, and synaptic proteins, and reduced ROS content without impacting hippocampal function. Opposite of what was expected, the South Beach diet, composed of high protein and high unsaturated fat, shows a severe pro-aging effect at the molecular level, promoting oxidative damage, increasing synaptic proteins related to excitotoxicity, and reducing the levels of ATP in the hippocampal cells, finally leading to learning and memory impairments. However, this is accompanied by increased levels of an antioxidant enzyme, fission–fusion-related proteins, and pre- and post-synaptic proteins, possibly in response to the activation of compensatory mechanisms against the damage induced by the South Beach diet. Also, this could explain the absence of more severe spatial memory impairment.

We show that feeding the Cocoa diet increases body weight, on average, by 2.03 g and reduces daily intake and caloric intake concerning the Control diet feeding at the end of the treatment period. A previous study demonstrates that a 60% fat diet induces body weight gain in SAMP8 mice [51] and C57Bl/6J [52]. Surprisingly, the South Beach feeding diet does not affect body weight but shows changes in daily consumption and caloric intake relative to the Control diet. The opposite effect was observed in Wistar rats fed high protein, such as South Beach feeding for 2.5 months, which increased their body weight relative to the Control diet [53], and in C57Bl/6J mice [54]. Our results show that the South Beach diet feeding resulted in a change in body weight compared to the high-carbohydrate Control diet over the three months of treatment, possibly due to lower food consumption and caloric intake. Following the diets, other important metabolic and health parameters will be affected, and future studies will be necessary to evaluate the concentrations of the different lipids, including saturated fat, MUFAs, PUFAs, and cholesterol, or lipoproteins, LDL, and HDL, and the levels of ketone bodies in the blood. Nevertheless, research has shown that high-protein diets can reduce LDL cholesterol levels, while high-fat diets can improve HDL cholesterol levels and reduce triglycerides. They may also increase LDL cholesterol, which could concern cardiovascular health [55,56]. Also, high-protein diets can enhance insulin sensitivity and glucose metabolism, reducing the risk of type 2 diabetes or metabolic syndrome [55]. In contrast, a high intake of saturated fatty acids significantly impacts lipid profiles in the blood, increasing the risk of cardiovascular diseases, increasing LDL cholesterol, total cholesterol, and triglycerides, and reducing HDL cholesterol [57,58]. More importantly, consuming PUFAs instead of saturated fat reduces coronary heart disease and its comorbidities [2,59]. Thus, high-fat and high-protein diets have unique benefits and potential drawbacks. The choice between them should be tailored to individual health goals, preferences, and metabolic needs [60].

Also, we report herein that SAMP8 mice fed the South Beach diet exhibited increased levels of 4-HNE in hippocampal lysates without increased levels of ROS. 4-HNE is one of the main peroxidation products and is an essential biomarker for oxidative brain damage and cerebral dysfunction in aging-associated diseases [30,37,61]. Conversely, our findings revealed reduced levels of ROS in the hippocampus of SAMP8 mice fed the Cocoa diet without effects on oxidative damage. These conflicting outcomes, juxtaposed with the observed 4-HNE levels, may find an explanation in the literature, where numerous studies indicate that a high-carbohydrate diet feeding can stimulate ROS generation in various brain and tissue cell types [62]. Nonetheless, it is vital to acknowledge that oxidative stress triggered by ROS plays a pivotal role in cellular and mitochondrial dysfunction [62]. At the same time, controlled levels of ROS also serve as a crucial component in cellular signaling pathways [63,64]. In addition, we evaluated the effect of diet on several antioxidant enzymes in the hippocampus. Our results show that the catalase levels were higher in mice fed the South Beach diet than in mice fed other dietary regimens. Catalase is crucial in the immediate antioxidant defense mechanism [65]. The heightened presence of this essential antioxidant component, induced by a diet rich in protein and unsaturated fat, may imply a potential response to the oxidative damage observed in the hippocampus of these mice. Additionally, we delved into the impact of diet on signaling pathways as an antioxidant response by examining Nrf2 and PGC-1α. Despite the high sensitivity of these factors to oxidative stress, we did not observe any discernible effects of these diets on the antioxidant response [66]; however, we only measured the total levels of the whole hippocampal lysate. The evaluation of Nrf2 and PGC-1α in the nucleus and mRNA of its target genes will be necessary. This unexpected outcome piques our interest and calls for further investigation into the complex interplay between diet and antioxidant response.

Fission and fusion processes regulate the mitochondrial network, which is crucial for maintaining the morphology and responding to various stress conditions induced by nutrient challenges within the cell [33,67]. Critical regulators in the fusion processes are Opa1 and mitofusins (Mfn1 y Mfn2). In fission, Drp1 and MFF participate. All these proteins respond to metabolic challenges resulting from the change in nutrients and allow the mitochondria to adapt to the energy requirements of the cell [68,69]. A study on the hippocampus of 8 mo SAMP8 mice revealed a reduction in the protein levels of Mfn2 and Opa1 for fusion and Drp1 for fission. These findings suggest a diminished capacity for fusion and fission processes within the hippocampus of aged SAMP8 mice. However, it is worth noting that this study did not specify the type of diet used in mice feeding [70]. Our results demonstrate that the South Beach diet feeding increases Opa1 levels in the hippocampus of SAMP8 mice compared to Control and Cocoa diet feeding. Opa1 promotes mitochondrial fission but also plays a crucial role in remodeling mitochondrial cristae [71], suggesting that during fission, Opa1 will play a protective or compensatory role for the formation and remodeling of cristae, thus contributing to the proper maintenance of mitochondrial bioenergetics. Furthermore, we observed increased fission proteins in the South Beach diet-fed mice, such as Drp1, which oligomerizes and activates Drp1 for mitochondrial fission and in its phosphorylation at serine 616 (pS616Drp1), which stimulates Drp1 function [25]. MFF, which is necessary for Drp1 translocation to mitochondria [58], did not show significant changes in its protein levels in hippocampal lysates after feeding the South Beach diet. These findings will suggest increased fission with the South Beach diet feeding. The literature does not describe whether a high-protein diet modifies the expression of proteins related to fission and fusion processes. However, it has been observed that diets rich in saturated and polyunsaturated fatty acids primarily increase fission and fusion, respectively [36]. These changes in fusion and fission proteins simultaneously allow us to hypothesize that increased fusion and fission processes may be occurring to compensate for or prevent the stress caused by the South Beach diet. Further studies must confirm these observations that propose increased mitochondrial fission and fusion processes or cristae remodeling.

Also, we investigated the influence of diets on the energetic state of the hippocampus in SAMP8 mice. Surprisingly, our analysis uncovered no differences in OXPHOS complexes across the dietary groups. These findings contrast with prior research suggesting that OXPHOS complexes may exhibit alterations in response to different nutritional regimens [72,73]. Specifically, many studies have reported significant decreases in complex I and III activities in mice subjected to a high-fat diet compared to those on a Control diet [73]. However, it is worth noting that these inconsistencies may stem from variations in the composition of dietary fats utilized. Our study employed both saturated and unsaturated high-fat diets, which could introduce distinct metabolic effects compared to studies utilizing different fat compositions. We investigated whether dietary interventions affected the levels of the critical proteins involved in mitochondrial bioenergetics, specifically on the levels of ATP synthase protein subunits such as ATP5a and OSCP. OSCP (F1Fo ATP synthase in mitochondria) regulates enzyme function and also forms a part of the mitochondrial permeability transition pore (mPTP) through its interaction with the Cyp-D [74]. Indeed, it is interesting that in the aging mouse brain and in various neurodegenerative diseases, a decrease in OSCP protein levels has been reported, correlating with reduced ATP production [39,40]. In our study, we observed a similar phenomenon wherein the decrease in OSCP protein levels in mice fed the South Beach diet was accompanied by reduced ATP levels in the hippocampus, proposing that OSCP reduction will lead to reduced mitochondrial function and energy production. Thus, decreased ATP levels in the hippocampus will be due, almost in part, to the reduced function of ATP synthase. Finally, these changes observed in fission and fusion proteins will also be due to the decreased energy state in the hippocampus of the South Beach diet-fed mice. More studies are necessary to validate these ideas.

Synaptic proteins are essential to neuronal communication and cognition, but their levels decrease with aging [42,75]. Therefore, to assess the impact of diet on the onset of the aging process in SAMP8 mice, we evaluated pre-synaptic, post-synaptic, and synaptic-function-related protein levels. We observed mainly increased proteins of the three categories mentioned previously in mice that were South Beach diet-fed. The South Beach diet is a high-protein diet with a high percentage of glutamic acid, which may promote an increase in the levels of glutamate, the principal neurotransmitter in hippocampal neurons [76]. This could lead to increased expression of synapsin, a protein responsible for transporting vesicles with neurotransmitters such as glutamate [44], NMDA receptors such as NR2B, which contributes to long-lasting synaptic plasticity [77], and Homer, a scaffold protein that enables glutamate receptor assembly and facilitates synaptic signaling [78], which together could compensate for the synaptic signaling and achieve consolidate memory. However, increased glutamate and other excitatory amino acids are associated with neurotoxicity [79], which may suggest that a high-protein diet, such as the South Beach diet, may promote modifications in synaptic protein expression and, thus, affect the cognitive level, which we observed in the MWM test. To determine the mechanisms involved in these protein changes, it would be interesting to evaluate the transcription factors associated with the synthesis of synaptic proteins and also evaluate their degradation to validate whether their increase is due to an up-regulated expression or a decrease in their degradation and, consequently, whether an accumulation of defective synaptic proteins occurs, impairing their function.

Conversely, although several publications that have evaluated high-fat diets have shown that they harm hippocampus-dependent cognition [80,81], in our study, we observed that SAMP8 mice fed the Cocoa diet showed no changes in the synaptic proteins measured and minimal changes in their performance in the MWM test. This may be because this diet’s high cholesterol content may help promote beneficial processes for the system, such as the myelination of hippocampal neurons [82], and may maintain a neuronal activity similar to control mice. Additionally, SAMP8 mice exhibit a baseline tendency towards lipogenic metabolism, leading to elevated systemic triglyceride levels and liver fat accumulation [83]. Research has indicated that transcription factors and signaling pathways involved in fat metabolism can influence the expression of proteins like GluR1, thereby promoting synaptic plasticity [84]. These observations suggest that a high-fat diet like Cocoa may help maintain protein levels and functionality.

Finally, it is important to highlight that the absence of adverse effects on hippocampal memory after the Cocoa diet or of more severe effects on cognitive performance with the South Beach diet, considering the numerous molecular changes present in the hippocampus of these animals, which could be due diverse factors or the combination of them, including (1) the activation of compensatory mechanisms, countering the cell stress induced by the diet, including mitochondrial mechanisms such as increased mitochondrial dynamics, antioxidant response, and changes related to increased synaptic plasticity; (2) the length of the diet, because the duration of the diet could play a crucial role; short-term dietary interventions might not be sufficient to manifest cognitive changes, even if molecular alterations are evident, so longer dietary regimens might be necessary to observe significant cognitive effects [85]; (3) the occurrence of specific molecular changes because not all molecular changes directly translate to an impairment in cognitive performance; some molecular alterations might be more related to metabolic processes or other physiological functions that do not directly impact cognition [86,87]; (4) the sensitivity of the cognitive test applied, since the classical Morris Water Maze might not be sensitive enough to detect subtle changes; some tests might only reveal differences under more extreme conditions or with more prolonged dietary interventions [88,89], so in future studies, we will perform complementary cognitive tasks such as memory flexibility, a water maze protocol in which the animal learns a new location of the hidden platform every day, using episodic-like memory [90]; and (5) the age of the mice starting with diet treatment; adult non-aged mice might have more plasticity in their cognitive functions, potentially masking the effects of dietary changes; in contrast, older mice might show more pronounced cognitive differences due to accumulated damage or reduced compensatory mechanisms [85,91]. Therefore, additional studies could consider implementing the diet at a more advanced age. Finally, each of these factors, or their combination, could mitigate the effects of the diets and reduce their impact on the aging phenotype. However, these small molecular changes, which produce subtle changes in memory, could be silent effectors for a predisposition or act as risk factors for developing a more aggravated aging phenotype or age-related pathology over time. Our findings underscore the importance of considering the temporality of treatment with different diets in research studies. Several studies with high-fat or high-protein diets have observed cognitive impairment, often associated with deregulating transcription factors and signaling pathways [81]. However, these studies usually overlook the aging factor and the basal oxidative stress of SAMP8 mice [92]. Therefore, our results, although seemingly contradictory to the current literature, may reflect an intermediate process in adapting the mouse to the food source and its effects, at the systemic level, on the onset of aging.

## 5. Conclusions

Altogether, our results suggest that the atherogenic Cocoa diet, which is high protein, high saturated fat, and high cholesterol, does not modify mitochondrial and synaptic proteins, oxidative and energetic states, and hippocampal function without effects on the aging phenotype in SAMP8 mice. In contrast, the South Beach diet, composed of high protein and high unsaturated fat, impacts the onset of brain aging, remarkably increasing the redox damage and reducing the bioenergetic state of the hippocampal brain and reducing the hippocampus-dependent spatial function in SAMP8 mice, with a pro-aging effect that accelerates the appearance of the aging phenotype. Thus, our results demonstrated that diets usually considered good could have opposite effects in the face of the onset of aging.

## Data Availability

For all the data supporting the reported results, please contact the corresponding author of the study.
